# Evaluation of accuracy of platelet rich plasma preparation devices for cardiac surgery: A prospective, randomized, single blinded study

**DOI:** 10.1051/ject/2026004

**Published:** 2026-06-19

**Authors:** Larry Garrison, Daniel Nicks, Samantha Nicks, Jonah Green, Huong Pham, Steve Wysocki

**Affiliations:** 1 Franciscan Health Indianapolis Indianapolis Indiana USA

**Keywords:** Platelet rich plasma, Platelet-derived growth factors, Cellular concentration, Cardiac surgery

## Abstract

*Background:* Applying platelet rich plasma (PRP) to the sternum immediately prior to approximation has been shown to enhance wound healing, lower the incidence of sternal wound infections, reduce costs associated with treating these infections and decrease post-operative pain scores. Multiple investigations have reported device specific outcomes regarding PRP preparation yields from healthy volunteer blood donors, all with initial platelet counts in the normal range. What is missing from the literature is how accurately PRP preparations reflect device-specific yield target values, particularly under the clinical conditions encountered routinely in the cardiac surgery arena. *Methods*: The Magellan^®^ group (30 cases) and the Angel^®^ group (30 cases) comprised the two study groups (2 groups, 60 total cases, 120 samples total). Pre and post processing blood samples from each group were analyzed for platelet counts. Platelet count increases were assessed for accuracy when compared to a specific target. *Results*: Individual yields from each tested device demonstrated some degree of limited variability. However, both groups mean values achieved and slightly exceeded the target value of a six-fold increase; Magellan group (*M* = 6.58, SD = 1.33), Angel group (*M* = 6.31, SD = 0.93). *Conclusion*: Both devices, on average, appear capable of accurately preparing PRP to meet the specific target value of a six-fold increase over baseline under conditions routinely encountered in cardiac surgery.

## Introduction

The application of whole blood derived autologous platelet rich plasma (PRP) in cardiac surgery immediately prior to sternal approximation has been shown to decrease superficial and deep sternal wound infections, reduce their associated cost burden, to enhance soft tissue wound healing and decrease post-operative pain scores [1–4]. The mechanisms attributed to these functionally diverse PRP-based growth factors, which include both autocrine and paracrine effects and their associated cellular sources, have been reported extensively [5, 6].

Reports comparing PRP devices, and their respective yields that are suitable for processing the whole blood volumes associated with cardiac surgery are less pronounced in the literature. Particularly absent are the clinical reports reflecting real-life situations encountered in the cardiac surgery environment, such as highly variable initial platelet counts.

Degen et al., compared the PRP yields of five different commercially available devices using seven healthy volunteers as whole blood donors [7]. Their investigation focused on comparing the measured compositional concentrations of multiple components of whole blood, such as platelets, white blood cells, neutrophils, red blood cells and pH. These investigators found that while there were significant compositional differences among the devices they tested, platelet recovery rates were not significantly different.

Questions remained regarding the accuracy of these devices when a specific yield (multiple of baseline platelet count) was targeted under cardiac surgery clinical conditions. Specifically, unlike utilizing whole blood from healthy volunteers, device accuracy remains uncertain when the whole blood samples contain initial platelet counts that are highly variable.

At our institution, PRP is routinely prepared, activated by a mixture of thrombin (5,000 IU) and 5 mL 10% Calcium Chloride, and applied for each cardiac surgery procedure. As such, the aim of this investigation focused on determining if the yield of PRP preparations of two separate PRP processing devices would accurately meet a pre-programmed or pre-established target value under the conditions encountered in the cardiac surgery arena.

## Materials and methods

The IRB at Franciscan Health Indianapolis determined this research to be exempt (ID2315425-1). Data from 60 cases involving 3 surgeons were prospectively collected. Whole blood samples were taken from twenty cardiac surgery cases of each surgeon (10 sample pairs in each group to be processed by one of two different PRP processing devices) between May and September, 2024. Normal procedures for patient care were followed as per departmental guidelines and physician orders. Good clinical judgment was used consistent with best practices during cardiac surgery procedures and with The Patient Safety and Quality Improvement Act of 2005 (PSQIA).

Inclusion criteria were those cases in which adult (18 years or older) patients of >50 kg were scheduled to undergo coronary bypass surgery or open cardiac procedures involving surgical correction of one or more cardiac valves or combined valve/coronary procedures. Exclusion criteria were patient age less than 18 years, pregnant females, emergent cases and any case in which the total whole blood processing volume was predicted to be or resulted in less than 180 mL.

The devices used in PRP preparation at our institution are the Angel^®^ cPRP System (Arthrex Inc., Naples, FL, USA), 7% Hct protocol, and the Magellan^®^ Autologous Concentration System (Isto Biologics Hopkinton, MA, USA), 6 mL volume protocol. For each case, a coin toss was used to randomize which device would be used to process the whole-blood samples. Regardless of device used, three 60 mL syringes were prepared by drawing 8mLs Anticoagulant Citrate Dextrose Solution-A (ACD-A) into each syringe. Each syringe was then capped with a sterile cap until the central line was placed as vascular access.

Once vascular access was achieved, 52 mL of whole patient blood was drawn from the central line catheter into each syringe to reach but not exceed a total volume of 60 mL. Prior to placing the filled syringes on a blood rocker, each syringe was gently inverted several times to ensure a thorough mixing of the whole blood with the citrate. A 500 μL sample from the last syringe to be filled was placed into a pediatric lavender blood tube and placed on the blood rocker.

For processing, the manufacturer's instructions for preparing 18 mL of PRP were followed for each device for achieving a 6× (six times) baseline platelet count yield. If the Magellan^®^ was used and upon completion of the PRP program, all three 10 mL syringes with 6 mL PRP each were dispensed into a 20 mL syringe. If the Angel^®^ was used and upon completion of the PRP program, enough platelet poor plasma (PPP) was drawn into the PRP syringe to reach a total volume of 18 mL.

Each PRP syringe was then gently inverted no fewer than 5 times to ensure a homogenous mix. Once a thoroughly mixed, a 500 μL sample of the final PRP product was placed into a pediatric lavender blood tube. Both the pre and post processing lavender blood tubes were then walked to the central laboratory for platelet count testing. A DxH 900^®^ (Beckman Coulter, Brea, CA, USA) analyzer was used to provide both pre and post platelet count results, which were then emailed to the principal investigator. For a detailed description of platelet count methodology, please see the DxH 900^®^ Instructions for Use [8]. The personnel at the hospital central laboratory were blinded as to which device had prepared the PRP. Data were analyzed with Excel^®^ (Microsoft, Redmond, WA, USA) and statistics were prepared with GraphPad Prism^®^, 10.4.2 (Graph Pad Software, Boston, MA, USA).

## Results

All platelet counts are presented as 10^3^ per μL. The yield multiple for each of the 60 pre and post platelet count sample pairs were calculated and the descriptive statistics for each group are listed in Table 1. An unpaired, two-tailed *t*-test was conducted to compare between group yield multiples in the Magellan^®^ group (*M* = 6.58, SD = 1.33) and the Angel^®^ group (*M* = 6.31, SD = 0.93). No significant difference were noted between the two groups, *t*(58) = 0.93, *p* = 0.35. Additionally, an *F*-test to compare variances between the values in each group was performed, which also was not significant: *F*(29, 29) = 2.07, *p* > 0.05 (Table 1).

**Table 1:** Device descriptive statistics.

Device	Mean platelet count (10^3^ per μL)	Median platelet count (10^3^ per μL)	Standard deviation	Standard error	Minimum Yield multiple (× baseline)	Maximum yield multiple (× baseline)	Device group range (× baseline)
Magellan	6.58	6.74	1.33	0.24	4.03	9.76	5.73
Angel	6.31	6.11	0.93	0.17	4.42	8.22	3.80

## Discussion

Perfusionists rely on the devices they use to produce the results or products that the manufacturers of these devices claim they will produce. As can be seen in Table 2, 50% of all Magellan^®^ device yield multiples fell between 5.72× baseline and 7.36× baseline, with a mean value of 6.58× baseline. The Angel^®^ produced similar results with 50% of all yields falling between 5.69 and 6.87, with a mean value of 6.31.

**Table 2:** Below normal initial vs yield platelet counts.

Angel Pre 10^3^ per μL	135.2	122.2	114.5	136.1	132.5	95.4	104.5	134.8	138.9	140	131.4
Angel Post 10^3^ per μL	966	752	754	829	796.8	633.6	537.5	892.5	733.7	792	891.3
X Baseline	7.14	6.15	6.59	6.09	6.01	6.64	5.14	6.62	5.28	5.66	6.78
Magellan Pre 10^3^ per μL	108.5	91.9	131.8	118	102	149.5	124.6	69.1	119.9	138.9	130.5
Magellan Post 10^3^ per μL	629.3	664.5	647	634.5	683	921.5	930.8	478.5	811.8	962.7	850.5
X Baseline	5.80	7.23	4.91	5.38	6.70	6.16	7.47	6.92	6.77	6.93	6.52

Interestingly, there were 11 samples in each group (36.7%) that had initial (pre) platelet counts of less than 150 × 10^3^ per μL, the lower limit of what is considered normal. Of those samples, three preparations in each group (27.3%) failed to achieve the target 6× baseline yield (Table 2).

Overall, there were 9 sample pairs in the Magellan group (30%) and 10 in the Angel group (33%) that failed to achieve the target 6× baseline yield (Table 3).

**Table 3:** Failed to achieve target yield.

Angel Pre 10^3^ per μL	168	222.4	219.7	104.5	189.7	151.1	251.7	256.2	138.9	140
Angel Post 10^3^ per μL	842	1178.8	1277.5	537.5	1081.4	815.1	1438	1132	733.7	792
X Baseline	5.01	5.30	5.81	5.14	5.70	5.39	5.71	4.42	5.28	5.66
Magellan Pre 10^3^ per μL	151.2	108.5	162	190.1	131.8	118	209.1	245.3	209.9	
Magellan Post 10^3^ per μL	657.5	629.3	946	765.4	647	634.5	996.2	1343.1	929.3	
X Baseline	4.35	5.80	5.84	4.03	4.91	5.38	4.76	5.48	4.43	

## Conclusion

There remains no consensus in the literature on the definition of what constitutes platelet rich plasma. Historically, the concentration of platelets above baseline values was the predominant characterization of the requirements to be considered PRP [9]. Since the time of that publication, there have been changes proposed to the definition, which would require an absolute platelet count minimum of 1 × 10^6^, though there are no official requirements to adhere to this practice [10]. In our study, 46.7% of all data pairs met the latter criteria. However, and irrespective of the definition, based on the results of our investigation, it would appear that both devices we tested, on average, produce a PRP product that achieved our 6× baseline target value.

The limitations of this study include being a single-blinded, single center investigation that focused only on targeting a single yield (6× baseline). Including additional investigational sites, utilizing a double-blinded protocol and targeting several different yields may be useful in providing further insight into the preparation accuracy of these devices.

## Data Availability

Original data is available by contacting the corresponding author.
